# Impact of adolescent ethanol exposure and adult amphetamine self-administration on evoked striatal dopamine release in male rats

**DOI:** 10.1007/s00213-015-4070-3

**Published:** 2015-09-26

**Authors:** L. Granholm, S. Rowley, M. Ellgren, L. Segerström, I. Nylander

**Affiliations:** Department of Pharmaceutical Biosciences, Division of Neuropharmacology, Addiction and Behaviour, Uppsala University, Uppsala, Sweden

**Keywords:** Chronoamperometry, Operant self-administration, Alcohol, Rodents

## Abstract

**Rationale:**

Adolescent binge drinking is common and associated with increased risk of substance use disorders. Transition from recreational to habitual ethanol consumption involves alterations in dorsal striatal function, but the long-term impact of adolescent ethanol exposure upon this region remains unclear.

**Objectives:**

This study aimed to characterise and describe relationships between adolescent ethanol exposure, amphetamine self-administration and adult dopamine dynamics in dorsal striatum, including response to amphetamine challenge, in male Wistar rats.

**Methods:**

Ethanol (2 g/kg) or water was administered intragastrically in an episodic binge-like regimen (three continuous days/week) between 4 and 9 weeks of age (i.e. post-natal days 28–59). In adulthood, animals were divided into two groups. In the first, dorsal striatal potassium-evoked dopamine release was examined via chronoamperometry, in the basal state and after a single amphetamine challenge (2 mg/kg, i.v.). In the second, amphetamine self-administration behaviour was studied (i.e. fixed and progressive ratio) before chronoamperometric analysis was conducted as described above.

**Results:**

Adolescent ethanol exposure suppressed locally evoked dopamine response after amphetamine challenge in adulthood, whereas in the basal state, no differences in dopamine dynamics were detected. Ethanol-exposed animals showed no differences in adult amphetamine self-administration behaviour but an abolished effect on dopamine removal in response to a single amphetamine challenge after self-administration.

**Conclusion:**

Amphetamine challenges in adult rats revealed differences in in vivo dopamine function after adolescent ethanol exposure. The attenuated drug response in ethanol-exposed animals may affect habit formation and contribute to increased risk for substance use disorders as a consequence of adolescent ethanol.

## Introduction

Adolescence represents a period of extensive reorganisation and maturation of brain circuits involved in emotions, motivation and cognition. This age is associated with high impulsivity, reduced behavioural control (Adriani and Laviola 2003; Arnett 1992; for review, see Spear 2000; Steinberg 2008) and altered risk valuation and decision making (Nasrallah et al. 2011; Nasrallah et al. 2009; Steinberg et al. 2009). These traits are commonly linked to increased risk for excessive drug consumption.

Alcohol is widely used among young people, and the consumption of the drug increases throughout adolescence (Swendsen et al. 2012). The age of first alcohol consumption is associated with both adult risk for alcohol use disorder (Dawson et al. 2008; DeWit et al. 2000; Grant and Dawson 1997; Hawkins et al. 1997) and substance use disorders (Anthony and Petronis 1995; Grant and Dawson 1998; Kandel et al. 1992; Yamaguchi and Kandel 1984). Thus, adolescence represents a window of particular susceptibility to alcohol exposure and its long-term implications.

In adolescence, a striatal-mediated increase in motivational drive for reward over prefrontal cognitive control leads to poor behavioural self-control compared to adults (Dahl 2008; Yurgelun-Todd 2007). The neuroanatomical pathway of motivation is complex but involves information flow through cortical–striatal pathways that can stimulate motor and behavioural outputs (Kalivas et al. 1999; Kolomiets et al. 2001; Masterman and Cummings 1997; Woodward et al. 1999). This information flow relies on extensive modulation from secondary motivational circuitry components to incorporate salient emotional, sensory and mnemonic inputs (Pennartz et al. 1994; Groenewegen et al. 1999). Within these networks, a basic compartmentalization of striatal function has been established, with reward, motivation and premotor cognition ascribed to the ventral region and behavioural initiation and habit formation to the dorsal striatum (Everitt and Robbins 2013; Vollstadt-Klein et al. 2010). It is suggested that alcohol use could disrupt this process and lead to long-term maladaptation that underlies the increased propensity to develop substance use disorders (Badanich et al. 2007; Guerri and Pascual 2010; Maldonado-Devincci et al. 2010; Sahr et al. 2004). However, whilst it has been established that dorsal striatal dopaminergic transmission is integral to the transition from recreational to habitual drug intake, the long-term impact of adolescent alcohol exposure upon this region remains unclear. Furthermore, the impact of adolescent ethanol exposure on adult self-administration of drugs of abuse other than ethanol and on drug-induced effects on adult dopamine dynamics is unknown.

Our hypothesis is that episodic binge-like ethanol exposure, typically present in adolescence, induces long-term neurobiological and behavioural alterations in brain regions and systems implicated in development of substance use disorders. In a recent study from our laboratory, the impact of voluntary adolescent ethanol drinking on in vivo dopamine dynamics in adult rats with and without a single amphetamine challenge was investigated (Palm and Nylander 2014). The results confirmed age-dependent basal and amphetamine-induced evoked dopamine release in ethanol-naïve animals, and in ethanol-drinking animals, a lower basal (i.e. potassium-induced) release was found, whereas the response to a single amphetamine challenge was unaffected (Palm and Nylander 2014). However, the animals were single housed during the voluntary drinking period, and it was of further interest to examine the pharmacological effects of ethanol on dopamine dynamics without the possible confounding factors of single housing (Meyer and Bardo 2015) and variations in the consumed amount of ethanol. Therefore, the specific aim in the present study was to investigate the long-term effects of episodic binge-like ethanol exposure (i.e. three consecutive days per week) using the same ethanol dose in all animals. Ethanol was administered orally in the same animal strain and sex as in the previous study, i.e. male non-alcohol preferring outbred Wistar rats. One group of animals was used to characterise adult basal and amphetamine-induced dopaminergic dynamics in the dorsal striatum by chronoamperometric recordings. A second group of animals was used to observe the influence of adolescent ethanol intake upon adult intravenous amphetamine self-administration behaviour. Subsequent chronoamperometric characterisation of these animals allowed group comparisons between amphetamine intake and dorsal striatal dopamine dynamics to be examined. Through these methods, we have demonstrated that binge-like adolescent ethanol exposure causes in vivo alterations in adult dorsal striatal dopamine dynamics as evident by altered response to amphetamine challenge.

## Materials and methods

### Animals

All animal experiments were performed under approval of the Uppsala Animal Ethical Committee and following the principles of the Guide for the Care and Use of Laboratory Animals and the guidelines of the Swedish Legislation on Animal Experimentation (Animal Welfare Act SFS1998:56) and the European Communities Council Directive (86/609/EEC).

**Fig. 1:**
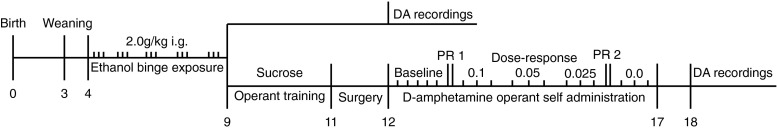
Experimental outline showing the time points (weeks) for the behavioural and neurochemical analyses. Dopamine (*DA*) recordings were done in vivo with high-speed chronoamperometry. *PR* represents progressive ratio sessions with two different doses PR1 (0.1 mg/kg/infusion) and PR2 (0.05 mg/kg/infusion), 52 × 14 mm (300 × 300 DPI)

Pregnant female Wistar rats (RccHan: WI, gestation day 16) were sourced from Harlan Laboratories B.V. (Horst, The Netherlands) and single housed under standard conditions (22 °C, 50 ± 10 % humidity, 12 h light–dark cycle commencing at 06:00, ad libitum access to pellet food and tap water, masking background noise). This is the least sensitive phase during pregnancy and was chosen to minimise the influence of stress related to transit. No signs of negative impact from transport were noticed during acclimatisation in the animal facility, and the delivery was normal in all females. The litters were cross-fostered and mixed so each litter contained four female and six males. Only male offspring were used in the continuation of the experiment. Upon weaning (post-natal day (PND) 21), animals were group housed under standard conditions as described above. Treatment groups were randomised, and adolescent animals (PND 28) were exposed to either ethanol or water. Upon completion of adolescent treatments (PND 59), two divergent protocols were followed, detailed schematically in Fig. 1.

### Drugs and solutions

Ethanol, Solveco Etanol A 96 % (Solveco AB, Rosersberg, Sverige), was diluted in tap water and d-amphetamine sulphate (Sigma-Aldrich, LLC, St. Louis, MO, USA) and diluted in sterile NaCl 9 mg/mL (Braun Melsungen AG, Melsungen, Germany). Sucrose food pellets (5-TUL, TestDiet, St. Louis, MO, USA) were used during the operant training. Anaesthesia agents were thiobutabarbital (Sigma-Aldrich, LSS, St. Louis, MO, USA), isoflurane (Forene Abbott, Solna, Sweden) or propofol (Braun Melsungen AG, Melsungen, Germany). For in vivo chronoamperometry, l-ascorbic acid, potassium chloride, sodium chloride, sodium phosphate and calcium chloride were purchased from Sigma-Aldrich, LLC (St. Louis, MO, USA) and diluted in Milli-Q water. For the post-operative care, buprenorphine (Schering-Plough, Brussels, Belgium), carpofen (Pfizer, Oy Animal Health, Helsinki, Finland) and amoxicillin (Ceva Animal Health, Dublin, Ireland) were used. For catheter maintenance, Heparin LEO (LEO Pharmaceuticals, Copenhagen, Denmark), sterile water (Braun Melsungen AG, Melsungen, Germany) and glycerol (Glycerol Unimedic AB, Matfors, Sweden) were used.

### Ethanol exposure

The animals received intragastric administration of either water or ethanol (2 g/kg, 20 % *w*/*v* ethanol diluted with water) for three consecutive days per week. This drinking paradigm was chosen to mimic common episodic adolescent drinking patterns and because intermittent ethanol exposure with drug-free days in-between has been shown to be necessary to induce neurobiological alterations similar to those seen in the transition to habitual and compulsive drinking (Spanagel 2003). The dose and route of administration were chosen to achieve binge-like oral consumption according to the National Institute on Alcohol Abuse and Alcoholism (NIAAA) definition of binge drinking (>0.08 g/dl in 2 h) (NIAAA Drinking Levels Defined). Unpublished results from other experiments in our laboratory as well as in published data from others (Walker and Ehlers 2009) have shown that 2 g/kg (intragastric) ethanol will produce blood alcohol concentrations reaching the binge criterion. Administrations were given at 09:00 on PND 28–30, 36–38, 43–45, 50–52, 57–59, followed by four days without treatment.

### In vivo dopamine recordings

For animals that underwent ethanol exposure only (ethanol *n* = 9, water *n* = 8), dopamine recordings were taken between 11 and 12 weeks of age (corresponding to the age of the initiation of amphetamine self-administration for the second set of animals). For the animals that additionally underwent self-administration trials, dopamine recordings were conducted during weeks 18 and 19 (ethanol *n* = 5, water *n* = 4). In all cases, animals were drug-free for a minimum of 2 weeks before dopamine recordings commenced (Fig. 1).

Dopamine recordings were conducted using carbon fibre microelectrodes (SF1A, 30 μm outer diameter, 150 μm length, Quanteon, LLC, Nicholasville, KY, USA). A high-speed chronoamperometric protocol was utilised (550 mV, 1 Hz sampling rate, 200 ms total) via a FAST16-mkII recording system (Quanteon). Electrode-pipette assemblies were prepared and calibrated immediately prior to in vivo recordings as previously described (Gerhardt and Hoffman 2001; Littrell et al. 2012). Briefly, electrodes were coated with Nafion (Sigma-Aldrich, LLC, St. Louis, MO, USA) and calibrated to cumulative additions of ascorbic acid and dopamine (ascorbic acid 250 μM, dopamine 2 μM steps) applied to a bath of 0.05 M phosphate-buffered saline. Electrodes used displayed a detection limit of 0.0237 ± 0.0037 μM and a selectivity of 3,864.35 ± 881.16 for dopamine over ascorbic acid. Responses to dopamine were linear, with an average correlation coefficient (*R*^2^) of 0.827 ± 0.025 and an average reduction/oxidation ratio of 0.628 ± 0.011 that is indicative of specific dopamine detection (Gerhardt and Hoffman 2001). After calibration, a micropipette filled with isotonic potassium chloride solution (120 mM KCl, 29 mM NaCl, 2.5 mM CaCl_2_, pH 7.2–7.4) was affixed with the tip 150–200 μm from the recording site of the electrode.

Animals were anaesthetized via intraperitoneal injection of 125 mg/kg thiobutabarbital and body temperature maintained with a thermostatic heating pad (Gaymar Industries, Inc., Orchard Park, New York). The electrode-pipette assembly was stereotaxically carefully placed in the dorsolateral striatum (AP +1.0, ML +3.0, DV −4.2 mm) and an Ag/AgCl reference electrode placed in the brain contralaterally and remote from working electrode-recording site.

After surgery and allowing 1 h for the stabilisation of electrode and surrounding tissue, 100 nl of potassium chloride solution was locally ejected using pressure ejection (PicoSpritzer III, Parker Hannifin Corporation, Pine Brook, NJ, USA; ejection pressure <22 psi, ejection time <2 s), and resultant local dopamine release was detected by the electrode as a peak of rising dopamine concentration. Potassium chloride ejections were repeated every 10 min until three successive consistent dopamine releases were recorded for use as baseline reference peaks. Five minutes after the third reference peak was evoked, a single 2-mg/kg dose of amphetamine was injected via tail vein. Five minutes post-injection of amphetamine, dopamine release was evoked again, and this was then repeated every 10 min until 55 min post-drug. Upon termination of recording, animals were sacrificed, then the brain removed and frozen for subsequent histological identification of electrode location.

### Self-administration

Rats from both the ethanol and water groups were subjected to assessment of self-administration behaviour followed by in vivo chronoamperometry (Fig. 1), the latter procedure is described above.

#### Operant chamber apparatus

Self-administration training and testing was conducted in sound-attenuated operant chambers (MED Associates Inc., Vermont, USA) equipped with two stimulus lights above two retractable stainless steel levers. A white house light placed on the wall opposite the levers was on during the entire session. A ventilating fan operated throughout the sessions and served as a masking noise. Intravenous solutions were delivered using an infusion pump (PHM-100, 3.33 rpm; Med Associates Inc.), and a 10-mL plastic syringe placed in the pump was connected to the implanted catheter through CoEx tubing (Harvard Apparatus, Kent, UK) and protected by a flexible metal leash (CamCaths, Ely, UK). Experiments were run and data collected by a PC with the MED-PC software (MedPC IV, MED Associates Inc., Vermont USA). The experiments were conducted in the same boxes for both training and test sessions, and ethanol- and water-treated animals were processed simultaneously throughout all phases of the self-administration procedure.

#### Sucrose training

Food restriction was initiated 48 h following the last intragastric pre-treatment session and was maintained throughout the self-administration training period to motivate food-seeking behaviour. Animal weights were carefully monitored and were not allowed to decrease more than 15 % from commencement of food restriction. After 2 days on food restriction, the training to self-administer 45 mg sucrose food pellets on a fixed ratio-1 (FR1) schedule was initiated. Each session started when the house light illuminated and the retractable levers were extended. A press on the active lever resulted in retraction of the lever and illumination of the stimulus light above the lever during a 10-s time-out period. The criteria for fulfilled self-administration training were accomplishment of 100 active lever presses within 30 min and a specificity >0.85 for the active lever.

#### Surgery

Intravenous catheters (CamCaths, Ely, UK) were implanted into the right jugular vein under isoflurane anaesthesia. Rats were administered post-operative analgesia (buprenorphine, 0.06 mg/kg s.c.; carpofen 5 mg/kg s.c.) and antibiotic (amoxicillin, 0.5 mL/kg s.c.). Catheters were flushed with a heparin solution (50 U/mL) before and after every session, and a heparinized glycerol lock solution (50:50 heparin/glycerol) was used over weekends. Catheter patency was tested before the start and at the end of the study with an infusion of the short-acting anaesthetic agent propofol.

#### Intravenous amphetamine self-administration

After a minimum of 4 days of recovery from surgery, rats (ethanol *n* = 8, water *n* = 8) were allowed to self-administer amphetamine on a daily 60 min FR3 schedule of reinforcement. Each session started when the house light was turned on and the retractable levers were extended. Three responses on the active lever resulted in an intravenous infusion of amphetamine (0.1 mg/kg/infusion), and a 10-s time-out period was initiated when both levers were retracted and a white stimulus light above the active lever was turned on. The lever designated to be the active lever was switched between sucrose training and intravenous self-administration. The maximum number of rewards during the 60-min baseline sessions was set to 20. A press on the inactive lever had no programmed consequences but was recorded by the software.

The rats underwent five baseline sessions of 60 min FR3 amphetamine (0.1 mg/kg/infusion) before the operant requirements were switched to the progressive ratio (PR) format. Under this schedule of reinforcement, the response requirement started at 1 and escalated for each drug infusion delivered according to following scheme: 1, 2, 4, 6, 9, 12, 15, 20, 25, 32, 40, 50, 62, 77, 95, 118, 145, 178, 219, 268, 328, 402, 492, 603, 737 and 901 (see Richardson and Roberts 1996). The PR schedule was tested at two doses (0.1 and 0.05 mg/kg per infusion) for two consecutive days each (see Fig. 1). The sessions ended when 1 h had passed since the last reward or after a maximum session time of 4 h. The breakpoint was defined as the total number of infusions during the session. Additionally, to test the dose–response function on a FR schedule, the unit dose of amphetamine (0, 0.025, 0.05 or 0.1 mg/kg per infusion) was varied, and each dose was tested for three consecutive 90 min FR3 sessions (Fig. 1). The first session of each dose in PR and dose–response trials was considered an acclimatisation session, and data collected during this session was not used in the statistical analysis.

### Data analysis

#### Dopamine analysis

The main parameters examined from dopamine oxidation currents, i.e. the peak area, the maximal amplitude (μM) of evoked peaks and the time taken for dopamine concentration to decline to 20 % of the maximum for each peak, T80 (seconds), were analysed with the FAST analysis software (version 5.2; Quanteon, KY, USA). The amplitude is a measurement of dopamine release; T80 is the uptake measure, whereas the peak area encompasses both the dopamine release and reuptake of dopamine. The chronoamperometric recordings of amplitude and T80 allow analysis of both the release and reuptake inhibition action of amphetamine.

For comparison of reference peaks, the raw values of the above parameters for the first three consecutive consistent dopamine releases obtained were compared via a repeated measures ANOVA to examine the influence of time, adolescent treatment group and time-group effects (Statistica 10; StatSoft Inc., Tulsa, OK, USA).

For analysis of amphetamine challenge, mean baseline values of parameters were obtained from the three reference peaks for each animal and subsequent values described as a percentage of this baseline. Subsequently, repeated measures ANOVA were used to examine time, adolescent treatment group and time-group effects. Where time-group differences were observed (*p* < 0.05), the Tukey´s HSD post hoc test was applied.

#### Self-administration

Student’s *t* test was used to compare the PR trials and sucrose pellet operant training. The parameters tested during operant training were the number of days until the rats had fulfilled the training criteria and the specificity for the active lever (number of presses on the active lever/total number of lever presses) when the criteria were fulfilled. The analysis of amphetamine self-administration behaviour on FR schedules was done with repeated measures ANOVA.

## Results

### Dopamine recordings after adolescent ethanol exposure

#### Reference values

Adolescent ethanol exposure alone did not affect evoked dopamine dynamics in adult animals in the basal state as evidenced by no statistically significant main effect of treatment across the three reference peaks (repeated measures ANOVA). The measurements (mean ± SEM) in water controls and ethanol-exposed rats, respectively, were as follows: amplitude, 3.43 ± 0.54 and 2.62 ± 0.39 [*F* (1, 15) = 1.5, *p* = 0.23]; area, 42.0 ± 8.87 and 33.1 ± 6.57 [*F* (1, 15) = 0.66, *p* = 0.43]; and T80, 12.8 ± 0.74 and 13.4 ± 1.18 [*F* (1, 15) = 0.15, *p* = 0.70]. Further, there was no interaction effect between time and treatment; amplitude [*F* (2, 30) = 0.46, *p* = 0.63], area [*F* (2, 30) = 0.18, *p* = 0.83] and T80 [*F* (2, 30) = 0.043, *p* = 0.96].

#### Amphetamine response after adolescent ethanol exposure

Acute amphetamine administration (single dose, 2 mg/kg, i.v.) resulted in significant increases in evoked dopamine peak area over time [effect of time: *F* (8, 120) = 19.4, *p* < 0.001]. No main effect of adolescent ethanol exposure was found in the peak area [effect of treatment: *F* (1, 15) = 2.30, *p* = 0.15)]. A significant interaction effect was observed indicating different amphetamine response in the two adolescent treatment groups [time × treatment: *F* (8, 120) = 2.03, *p* = 0.048]. The post hoc analysis revealed that animals exposed to ethanol had a significant (*p* = 0.007) increased peak area 5 min after the amphetamine challenge, whereas the water-exposed animals had a prolonged increase in peak area with a statistically significant increase at 5 (*p* < 0.001), 15 (*p* < 0.001), 25 (*p* = 0.001) and 35 (*p* = 0.007) min after the amphetamine challenge.

**Fig. 2:**
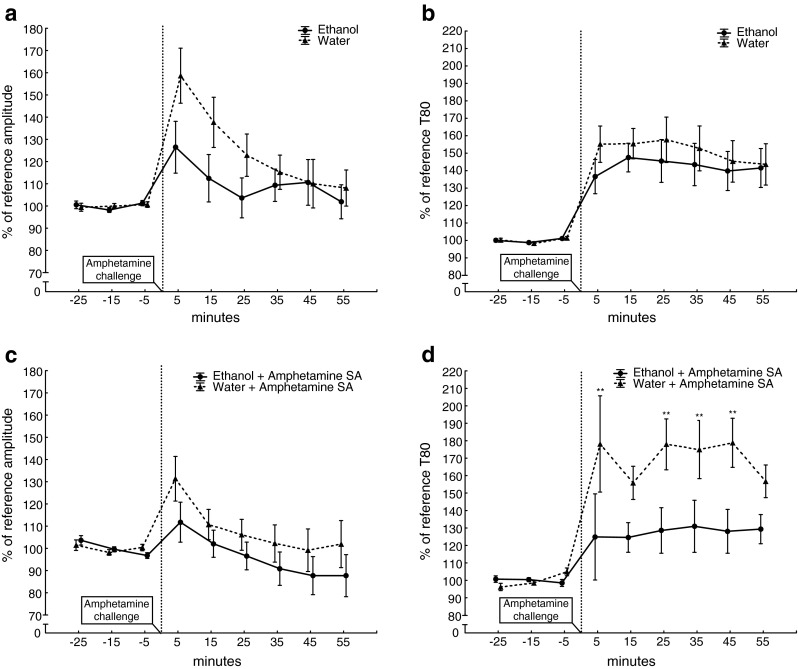
Amplitudes (**a** and **c**) and T80 (**b** and **d**) measurements over time after different drug exposures. The *graphs* show the responses after an intravenous amphetamine challenge (2 mg/kg i.v.) as percent (mean ± SEM) of reference values after adolescent ethanol (*n* = 9, *circles*) or water (*n* = 8, *triangles*) exposure (**a** and **b**) and after adolescent ethanol (*n* = 5, *circles*) or water (*n* = 4, *triangles*) exposure followed by amphetamine self-administration (**c** and **d**). **p < 0.01 compared to the time point (−5) before the amphetamine challenge, repeated measures ANOVA followed by Tukey’s HSD test, 150 × 131 mm (300 × 300 DPI)

Analysing the parameters included in the peak area revealed that the difference was mainly driven by an interaction effect in peak amplitude [time × treatment: *F* (8, 120) = 1.93, *p* = 0.061] (Fig. 2a). A main effect of time [*F* (8, 120 = 9.79 *p* < 0.0001] was found, but no main effect of treatment alone was seen in the amplitude [*F* (1, 15) = 1.63 *p* = 0.22]. In the T80 values, there were no main effect of treatment [*F* (1, 15) = 0.40 *p* = 0.53] or interaction effect [time × treatment: *F* (8, 120) = 0.50, *p* = 0.85] but a main effect of time [*F* (8, 120) = 26.96, *p* < 0.0001] (Fig. 2b).

### Effects of adolescent ethanol exposure and adult operant self-administration

#### Sucrose training

There was no between-group difference in the numbers of days until the rats had fulfilled their sucrose training (water controls, 6.4 ± 0.4; ethanol-exposed rats, 6.2 ± 0.4; *t* = −0.24, *p* = 0.81) or the specificity for the active lever (water controls, 0.94 ± 0.02; ethanol-exposed rats, 0.94 ± 0.01; *t* = −0.11, *p* = 0.91) at the last day of the sucrose training.

#### Amphetamine self-administration

**Fig. 3:**
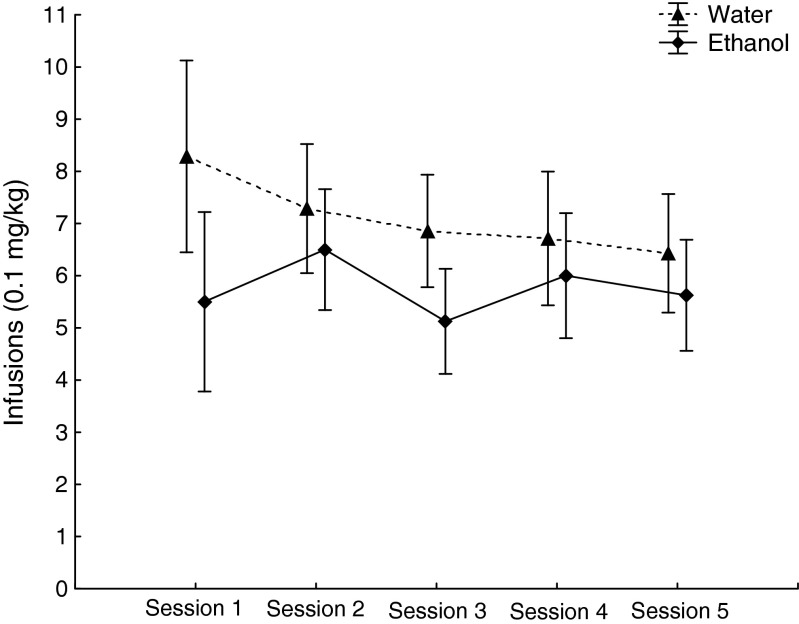
Acquisition of amphetamine self-administration. The values represent mean (±SEM) number of amphetamine infusions earned on a fixed ratio 3 schedule across five baseline sessions. Water (*n* = 8) and ethanol (*n* = 8), 90 × 70 mm (300 × 300 DPI)

**Fig. 4:**
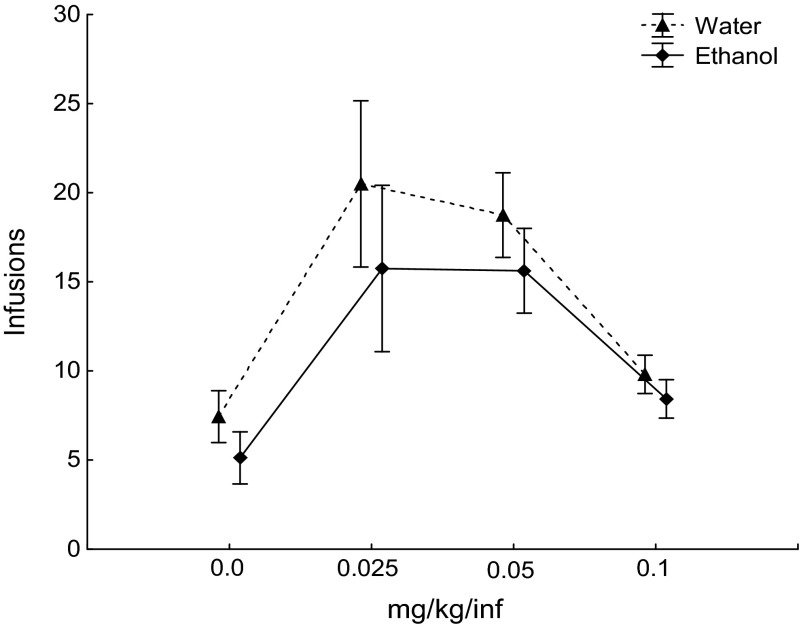
Dose–response function for amphetamine self-administration. The values represent mean (±SEM) number of amphetamine infusions earned on a fixed ratio 3 schedule across different unit doses. Water (*n* = 8) and ethanol (*n* = 8), 92 × 71 mm (300 × 300 DPI)

**Fig. 5:**
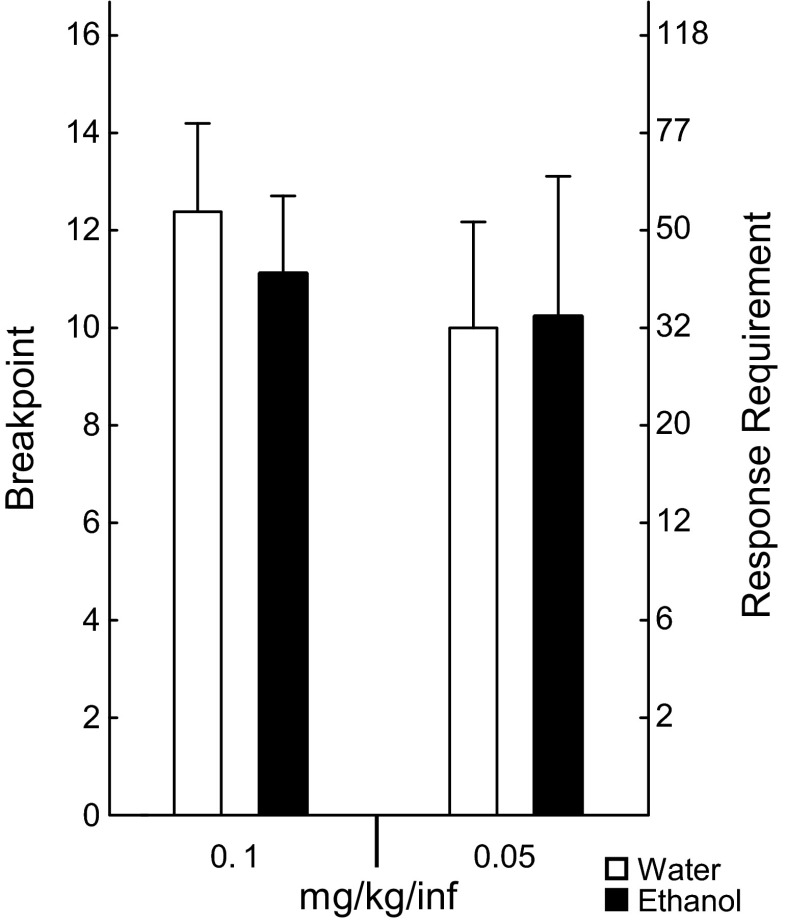
Breakpoint values (rewards earned during the trial) and corresponding response requirements for the last earned reward when amphetamine was self-administered on a progressive ratio schedule of reinforcement. Water (*n* = 8) and ethanol (*n* = 8). *Bars* represent mean + SEM, 83 × 97 mm (300 × 300 DPI)

Repeated measures ANOVA revealed no main effect of treatment [*F* (1, 13) = 1.65, *p* = 0.22] or interaction effect between treatment and session [(*F* (4, 52) = 0.29, *p* = 0.88)] in the five baseline sessions (Fig. 3). No main effect of treatment (*F* (1, 14) = 1.06, *p* = 0.32) or interaction effect between dose and treatment [*F* (3, 42) = 0.2, *p* = 0.89] was found during the FR schedule dose–response function (Fig. 4). The rat motivational drive to consume amphetamine was tested in PR trials at two different doses and shown in Fig. 5; the breakpoint value did not differ between the two groups at any dose: 0.1 mg/kg/infusion trial (*t* = 0.52; *p* = 0.61), 0.05 mg/kg/infusion trial (*t* = −0.070, *p* = 0.94).

### Challenge with amphetamine after adolescent ethanol exposure and adult amphetamine self-administration

#### Reference peaks

No main effect of treatment was observed in adult reference peaks after amphetamine self-administration (repeated measures ANOVA). The measurements (mean ± SEM) in water controls and ethanol-exposed rats, respectively, were as follows: amplitude, 2.86 ± 0.27 and 3.96 ± 0.42 [*F* (1, 7) = 1.25, *p* = 0.30]; area, 53.5 ± 7.65 and 76.1 ± 9.29 [*F* (1, 7) = 1.01, *p* = 0.34]; and T80, 17.8 ± 0.85 and 18.1 ± 0.44 [*F* (1, 7) = 0.04, *p* = 0.85]. Also, no interaction effect between time and treatment was observed: amplitude [*F* (2, 14) = 1.1349, *p* = 0.35], area [*F* (2, 14) = 1.97, *p* = 0.18] and T80 [*F* (2, 14) = 3.20, *p* = 0.07].

#### Amphetamine response after adolescent ethanol exposure and adult amphetamine self-administration

After self-administration, a single amphetamine challenge (2 mg/kg, i.v.) resulted in significant increases in evoked dopamine peak area over time [effect of time: *F* (8, 56) = 7.35, *p* < 0.00001], and significant differences were observed in amphetamine response between the two adolescent treatment groups [effect of treatment: *F* (1, 7) = 8.44, *p* = 0.023]. An interaction effect between time and treatment [*F* (8, 56) = 3.56, *p* = 0.0021] was found. The post hoc test showed that the ethanol-exposed rats did not increase their peak area after the amphetamine challenge, whereas water-exposed animals had an increased peak area 5 (*p* < 0.001), 15 (*p* = 0.02), 35 (*p* = 0.05) and 45 (*p* = 0.006) min after the amphetamine challenge.

Further analysis of the parameters included in the peak area revealed that the difference in peak area was mainly driven by the T80 values where main effects of treatment [*F* (1, 7) = 6.84, *p* = 0.035] and time [*F* (8, 56) = 10.67, *p* < 0.0001] as well as an interaction effect between time and treatment [*F* (8, 56) = 2.28, *p* = 0.035] were found (Fig. 2d). The reduced T80 in ethanol-exposed rats suggests a more efficient removal of dopamine. In the amplitude values, a main effect of time [*F* (8, 56) = 5.69, *p* < 0.0001] was found but no main effect of treatment [*F* (1, 7) = 1.36, *p* = 0.28] or interaction effect between time and treatment [*F* (8, 56) = 1.01, *p* = 0.44] (Fig. 2b).

## Discussion

The present study is, to the best of our knowledge, the first to investigate both in vivo dopamine dynamics in dorsal striatum as well as amphetamine self-administration after adolescent binge-like ethanol exposure. The study provides new evidence for long-term alterations in dopamine dynamics within the dorsal striatum. Specifically, the consequences of being exposed to ethanol during adolescence were revealed when the animals were subjected to amphetamine challenge in adulthood.

### Adolescent ethanol exposure and adult in vivo dopamine

The dopaminergic system in dorsal striatum undergoes significant changes during adolescence. Dopaminergic receptors (Gelbard et al. 1989; Giorgi et al. 1987; Teicher et al. 1995), tyrosine hydroxylase (Mathews et al. 2009; Matthews et al. 2013), dopamine transporters (Matthews et al. 2013; Truong et al. 2005) as well as the readily releasable and storage pool of dopamine (Stamford 1989) all differ between adolescents and adults. Therefore, the disruption of any aspect within this maturational process by repeated exposure of ethanol can be expected to impact dopamine function in adulthood.

The present data show that adolescent ethanol exposure had no effect on basal dopamine dynamics but resulted in reduced evoked peak area after amphetamine challenge in adult rats. Furthermore, the results indicate that suppressed dopamine release rather than more efficient dopamine removal contributed to this effect. Recent chronoamperometric studies conducted in our laboratory show that voluntary adolescent drinking significantly reduces basal (i.e. pre-amphetamine challenge) potassium-induced peak amplitudes in adulthood but did not affect dopamine responses to a single amphetamine challenge (Palm and Nylander 2014). The compiled results show that the impact of episodic adolescent ethanol exposure differs depending on ethanol intake paradigm. The discrepancies between the chronoamperometric results from the two studies can be a consequence of different study populations; the present study describes the effects of ethanol binges during adolescence independent of phenotype, whereas the results from Palm and Nylander (2014) describe ethanol effects in a drinking phenotype. The present study employed a forced administration regimen instead of voluntary drinking to be able to target the pharmacological effects of a pre-set (2 g/kg i.g.) dose of ethanol, independent of drinking patterns and intake. A limitation with the forced administration is stress induced by the gavage procedure. Stress activation affects the dopamine systems and could therefore be a potential confounding factor (Ungless et al. 2010). However, a recent study showed that intragastric administration had less impact on the HPA axis than intraperitoneal injections (Hoffman et al. 2014) which is in favour of the route used herein. On the other hand, voluntary drinking usually involves single housing which per se increases amphetamine self-administration in adulthood (Meyer and Bardo 2015) and would confound ethanol effects in the present experiment. Furthermore, many non-preferring rats do not ingest enough ethanol in a voluntary drinking model to achieve the BAC necessary enough for a “binge.”

Other studies using microdialysis in mesolimbic regions have shown that repeated ethanol exposure increases basal extracellular levels of dopamine in adolescent rats (Badanich et al. 2007; Pascual et al. 2009; Philpot et al. 2009), but these effects did not last into adulthood (Pascual et al. 2009). Also in line with our findings of lower dopamine response after a drug challenge, intermittent ethanol exposure (i.p.) during adolescence decreased accumbal ethanol-evoked dopamine release 7 and 14 days after treatment, but the effect was diminished after 28 days (Zandy et al. 2014). Furthermore, repeated treatment with ethanol in preadolescence (PND 21–24) or early adolescence (PND 31–34) lowered dopamine efflux in nucleus accumbens after ethanol challenge (Philpot et al. 2009).

### Adolescent ethanol exposure and adult amphetamine self-administration

It has previously been shown that a history of adolescent ethanol exposure increases subsequent voluntary intake of ethanol (Alaux-Cantin et al. 2013; Criado and Ehlers 2013; Pascual et al. 2009). However, the literature is not conclusive, several studies report subtle or no effect on subsequent intake (Criado and Ehlers 2013; Gilpin et al. 2012; Siegmund et al. 2005; Slawecki and Betancourt 2002; Vetter et al. 2007). Surprisingly, little work has investigated how adolescent ethanol exposure influences intake of drugs of abuse other than ethanol. With regard to the growing evidence that adolescent ethanol exposure affects the dopamine transmission, drugs that specifically target this system are of interest. It has been shown that ethanol exposure during early/mid-adolescence (PND 30–39) sensitised the rewarding effects of cocaine and attenuated the aversive effects as well as altered gross locomotor activity (Hutchinson and Riley 2012; Hutchison et al. 2010). The impact on amphetamine-induced reward is not known, and since amphetamine was used as a challenge drug in the present study, it was of interest to further assess the initial rewarding effects of amphetamine in the self-administration paradigm.

Since cognitive and behavioural dysfunction can be seen after adolescent ethanol exposure (for review, see Guerri and Pascual 2010), sucrose training was performed before initiating the amphetamine self-administration. No differences between the groups were found in any of the parameters tested during the sucrose training, indicating that the ethanol exposure had no effect on the learning ability for the operant procedure and did not affect the general motivation for a non-drug reward.

Analysis of the self-administration behaviour revealed that there were no differences between the groups in their initial response to amphetamine (i.e. baseline sessions). With the concept of a dorsal striatal involvement in the shift to habitual drug use (Everitt and Robbins 2013) in mind, we anticipated that there might be a difference between the groups motivational drive to consume amphetamine. However, no differences between water- and ethanol-exposed rats were found in the PR trials (i.e. 0.1 or 0.05 mg/kg per infusion). A dose–response function was used to further explore the self-administration behaviour on the FR schedule, but the groups displayed similar intake behaviour in all doses tested.

Our hypothesis, based on previous literature and the findings from our own experiments, was that dorsal striatal dopamine and amphetamine self-administration would be affected by adolescent ethanol exposure. The noted difference in peak area, driven by reduced release of dopamine (reduced amplitude) after amphetamine challenge in the ethanol-exposed group, had, however, no influence on drug-taking behaviour during the short period of self-administration used herein to examine initial drug reward. Nevertheless, there are a number of ways to modify the amphetamine self-administration paradigm, and our results might not be conclusive. A different setup with prolonged self-administration or a shift toward lower doses might reveal more subtle alterations between the groups. Amphetamine is a potent drug, and the doses used and the length of the exposure periods might overrule the effects of adolescent ethanol exposure. To observe specific subgroups in relation to adolescent influence upon drug intake and responding, specific inclusion criteria (e.g. a certain number of rewards per session) are commonly used. In the present study however, all animals displaying amphetamine self-administration behaviour were included, including the low responding animals. The ethanol exposure during adolescence was independent of phenotype, and we wanted to keep this heterogeneity throughout the experiment. Importantly, all animals included were familiarised with the operant technique (i.e. the sucrose training) before the initiation of amphetamine self-administration. In both the fixed and progressive ratio trials, large individual differences in amphetamine intake were also found that were independent of adolescent ethanol exposure.

### Adolescent ethanol exposure, amphetamine self-administration and in vivo dopamine

In rats, with or without a history of adolescent ethanol exposure, in vivo dopamine dynamics were analysed in the dorsal striatum 2 weeks after the last amphetamine self-administration session. The response to a single challenge with amphetamine was attenuated in animals with both adolescent ethanol exposure and repeated adult amphetamine self-administration as compared to those with only adult amphetamine intake. The reduced response was driven by an abolished effect on T80 (the time for removal) possibly indicating different effects on transporter function. Thus, even though no differences were noted in amphetamine intake in the self-administration assessment, the response to the drug in the brain was altered in ethanol-exposed animals. These results indicate potential for a synergistic effect upon dopaminergic response to amphetamine after adolescent ethanol and adult amphetamine exposure. An interesting aspect would have been to investigate how these rats responded to a reintroduction of amphetamine self-administration, for example another PR trial, lower unit doses of amphetamine or extended repeated periods of self-administration.

To summarise our findings, male rats exposed to ethanol in adolescence had, as adults, somewhat reduced dopamine release after a single amphetamine challenge but no differences in amphetamine self-administration with the current experimental setup. However, amphetamine challenge after adult amphetamine intake through self-administration revealed pronounced differences in striatal dopamine removal between animals with or without a history of adolescent ethanol exposure. Such difference in drug-induced dopamine response in ethanol-exposed individuals may affect their further drug-taking behaviour. If so, it can be speculated that a vulnerability for later excessive drug administration previously reported after adolescent ethanol exposure may not be seen at the first contacts with a drug in young adults but rather emerge with repeated drug use. However, this was not tested in the present study, and further studies are warranted to elucidate the relevance of the altered drug response reported herein for altered drug taking as a consequence of adolescent ethanol exposure as well as investigate possible differences between the sexes.

## Abbreviations

FR: Fixed ratio
PR: Progressive ratio
PND: Post-natal day
